# The potential of hematopoietic growth factors for treatment of Alzheimer's disease: a mini-review

**DOI:** 10.1186/1471-2202-9-S2-S3

**Published:** 2008-12-03

**Authors:** Juan Sanchez-Ramos, Shijie Song, Chuanhai Cao, Gary Arendash

**Affiliations:** 1 Dept of Neurology, University of South Florida, Tampa, FL 33612, USA; 2 Byrd Institute for Alzheimer's Disease Research, Tampa, FL, 33612, USA

## Abstract

There are no effective interventions that significantly forestall or reverse neurodegeneration and cognitive decline in Alzheimer's disease. In the past decade, the generation of new neurons has been recognized to continue throughout adult life in the brain's neurogenic zones. A major challenge has been to find ways to harness the potential of the brain's own neural stem cells to repair or replace injured and dying neurons. The administration of hematopoietic growth factors or cytokines has been shown to promote brain repair by a number of mechanisms, including increased neurogenesis, anti-apoptosis and increased mobilization of bone marrow-derived microglia into brain. In this light, cytokine treatments may provide a new therapeutic approach for many brain disorders, including neurodegenerative diseases like Alzheimer's disease. In addition, neuronal hematopoietic growth factor receptors provide novel targets for the discovery of peptide-mimetic drugs that can forestall or reverse the pathological progression of Alzheimer's disease.

## Hematopoietic growth factors influence neurogenesis

Hematopoietic growth factors (HGFs) enhance proliferation, differentiation, and release of blood cell lineages into the circulation. Interestingly, these same cytokines have been shown to influence the proliferation of neural stem/progenitor cells. Review of the similarities and differences between the stem cells that give rise to neural cell lineages ('neuropoiesis'), and those that generate the various blood cell lineages (hematopoiesis), has led to the concept of the 'brain marrow' [1,2]. 'Brain marrow' was conceived as a proliferative central core of the central nervous system comprising the subependymal zone and other cell groups (sub-granular zone of the hippocampal dentate gyrus) that give rise to new neural cells throughout life. In addition to requiring special extracellular matrix and supportive cells (the stem cell 'niches'), both sets of stem cells require trophic factors, cytokines, and other molecules that are instructive for proliferation, migration, and differentiation into their respective cell lineages. A major overlap between bone and brain marrow is seen in the collection of cytokines and trophic factors that promote blood cell and neural cell development.

Many of the cytokines and growth factors required for both hematopoeisis and neuropoiesis are constitutively expressed by bone marrow (BM) stromal cells [1,2]. Two cytokine superfamilies produced by BM stromal cells are transforming growth factor-β and the hematopoietins. They mediate a range of developmental events in the nervous system that rivals that of the classic neurotrophins. The bone morphogenetic proteins (BMPs), a sub-class of the transforming growth factor-β superfamily, are well known for their significant effects on the development of osteocytes and chondrocytes in bone. However, BMP ligands and their receptor subunits are also present throughout neural development within discrete regions of the embryonic brain and within neural crest-derived migratory zones [3,4]. BMPs exhibit a broad range of cellular and context-specific effects during multiple stages of neural development [5]. For example, BMPs initially inhibit the formation of neuroectoderm during gastrulation, while within the neural tube they act as gradient morphogens to promote the differentiation of dorsal and intermediate cell types through co-operative signaling. BMP-2 acts synergistically with fibroblast growth factor (FGF2) on more lineage-restricted embryonic central nervous system progenitor cells to induce the expression of the dopamine neuronal marker tyrosine hydroxylase [6]. Various hematopoietic cytokines have been shown to enhance the number of dopaminergic neurons in mesencephalic cultures, but only interleukin-1 induced the expression of the dopamine neuronal marker tyrosine hydroxylase in the progenitor cells [7].

Other regulatory proteins that influence blood cell differentiation have effects on neuronal development. Erythropoietin, in addition to its function to promote the production of red blood cells, also has trophic effects on central cholinergic and cortical neurons [8]. Erythropoietin receptor mRNA is expressed in the mouse brain and in the central nervous system of the developing human fetus. Neuronal cell lines, including PC12 and SN6, also express a functional erythropoietin receptor. The neurotrophic activity of erythropoietin has been demonstrated in neural cells [9]. A 17-mer peptide sequence (EPO peptide) in erythropoietin has been identified that has activity similar to that of the holoprotein [9]. This peptide induced differentiation and prevented the proliferation of erythropoeitic cell lines and mouse primary spleen cells. When the EPO peptide or erythropoietin was locally injected into mice, the frequency of motor end plate sprouting in adjacent muscles increased in a manner similar to that induced by ciliary neurotrophic factor. Since neural cells, but not hematological cells, respond to a specific peptide sequence within erythropoietin, it is likely that the holoprotein has separate domains for neurotrophic and hematotrophic functions.

Neural stem/progenitor cells of the brain express c-*kit*, the receptor for one of the major hematopoietic cytokines, stem cell factor (SCF). In hematopoietic cells, SCF and *c-kit *regulate a variety of developmental events, including cell proliferation, survival and differentiation [10]. SCF has also been shown to stimulate neurogenesis both *in vitro *and *in vivo *[11]. Not only is *c-kit *expressed in neurogenic zones of the brain, but ischemia has been reported to alter *c-kit *expression, which in turn stimulates neural stem cell (NSC) proliferation and differentiation into neurons [11]. The interaction of SCF with its receptor *c-kit *on NSCs also appears to play a key role in triggering NSC migration from the neurogenic zones to sites of brain injury [12].

Granulocyte-colony stimulating factor (G-CSF) is a potent growth factor that stimulates proliferation of hematopoietic stem cells and drives differentiation of myeloid lineage cells. It is used extensively in clinical practice to accelerate recovery of patients from neutropenia after cytotoxic therapy. G-CSF also impacts neural tissues [13]. When administered systemically, it crosses the blood-brain barrier [14]. The G-CSF receptor and its ligand are expressed by neurons in a variety of brain regions, including pyramidal cells in cortical layers (particularly in layers II and V), Purkinje cells in the cerebellum, the subventricular zone and in cerebellar nuclei in rats [14]. G-CSF positive cells have been identified in the CA3 region of the hippocampus, the subgranular zone and hilus of the dentate gyrus, entorhinal cortex, and the olfactory bulb [14]. G-CSF receptor expression has also been shown in the frontal cortex of human brain by postmortem studies [14].

Both neuronal G-CSF and its receptor are up-regulated in the ipsilateral forebrain hemisphere 2 hours after the occlusion of the middle cerebral artery and reperfusion in a rat model of stroke [14]. In a similar study, G-CSF mRNA levels were massively increased by ischemia compared to normal cortex and returned to control levels after 2 days [15]. The increase in G-CSF mRNA expression extended beyond the ischemic lesions to include non-ischemic frontal cortex after photothrombosis in a rat model of focal cerebral ischemia [15]. In a mouse model of stroke, administration of the combination of G-CSF and SCF 10 days after ischemia effectively improved motor performance, induced transition of BM-derived neuronal cells into the peri-infarct area, and stimulated proliferation of intrinsic neural stem/progenitor cells in the neuroproliferative zone [16]. It has been suggested that G-CSF may have a protective autocrine signaling mechanism in response to neural injury, similar to a mechanism suggested for other growth factors, especially erythropoietin [17].

## Bone marrow-derived cells in Alzheimer's disease

A growing focus of research has been on the discovery of multipotent stem cells in BM that are capable of giving rise to tissues of all embryonic germ layers [18]. BM-derived cells have been shown by independent investigators to give rise to neural cells and these may migrate to brain where they appear to differentiate into neurons and glia [19-22]. The mechanism for transdifferentiation of BM to neural cells is not clear and may reflect the capacity of BM-derived cells to fuse with injured neurons [23].

The demonstration of transdifferentiation of BM cells has primarily been performed *in vitro*, utilizing different sub-populations of marrow, and various combinations of cell culture media and growth factors. The combination of retinoic acid and brain-derived neurotrophic factor (BDNF) was effective in driving a BM stromal cell population into neuron-like cells [19]. More recently, other researchers have reported that a fragment of amyloid precursor protein potentiates the nerve growth factor/retinoic acid-induced transdifferentiation of BM-derived adult progenitor cells into neural progenitor cells and, more specifically, enhances their terminal differentiation into a cholinergic-like neuronal phenotype [24]. *In vivo *work on the problem of transdifferentiaton suggests that generation of new neurons from a bone marrow progenitor, if it really occurs, may be a rare phenomenon. Nevertheless, BM cells from the periphery are the source of a small proportion of the brain's immune cells (microglia) in the normal adult brain, and following various brain injuries, the trafficking of BM cells from blood to brain is markedly increased.

Microglia have been clearly observed in the core of amyloid plaques in transgenic mouse models of Alzheimer's disease (AD). Perhaps as many as 10% of these cells originate from the BM, and the amyloid-β (Aβ)-40 and Aβ-42 isoforms are able to trigger this chemoattraction. These newly recruited cells also exhibit a specific immune reaction to both exogenous and endogenous Aβ in the brain [25].

Infiltration of the brain parenchyma by BM-derived cells in the course of cerebral amyloidosis has been demonstrated by tracking green fluorescent protein (GFP)-BM in the tg mouse model (APP23 mice) of AD [26]. These mice underwent lethal irradiation and were rescued by BM replacement with GFP-expressing BM cells (from a tg GFP mouse line). Interestingly, aging amyloid-depositing APP23 mice exhibited a significant increase in the number of invading GFP-positive cells compared with age-matched, non-transgenic control mice. Only a subpopulation of amyloid deposits was surrounded by invading cells, suggesting that not all amyloid plaques were a target for invading cells. Another possibility was that all amyloid plaques attracted infiltrating immune cells, but only for a limited time, possibly at an early stage of plaque evolution. Immunological and ultra-structural phenotyping revealed that macrophages and T cells accounted for a significant portion of these ameboid-like invading cells. Macrophages did not show evidence of amyloid phagocytosis at the electron microscopic level, and no obvious signs of T cell-mediated inflammation or neurodegeneration were observed. Hence, the observation that BM-derived cells invade the brain in response to cerebral amyloidosis may provide a novel therapeutic approach [26].

In another study, young transgenic mice were transplanted with GFP^+ ^BM cells to create chimeric mice before the onset of AD-like pathology and their brains were analyzed 6.5 months later. The number of engrafted BM-derived cells was significantly higher than in age-matched wild-type mice. Moreover, the number of BM-derived cells associated with Aβ was significantly higher than in older transgenic mice transplanted after the establishment of AD-like pathology. Local inflammation caused by intrahippocampal lipopolysaccharide injection significantly increased the engraftment of BM-derived cells in old AD mice and decreased the hippocampal Aβ burden. These results suggest that infiltration of BM-derived monocytic cells into the brain contributes to the development of a microglial reaction in AD [27].

Most recently, administration of G-CSF into two different Aβ-induced AD mouse models substantially improved performance on the Morris water maze [28]. In the 'acute' model of AD, aggregated Aβ was injected into the junction of the hippocampus and cortex of the C57BL/6 mouse brains. In the chronic model of AD, the investigators used Tg2576 mice, which overexpress the human amyloid precursor protein with the Swedish mutation. The administration of G-CSF also resulted in enhanced incorporation of bromodeoxyuridine by hematopoietic stem cells (HSCs), as well as local neurogenesis surrounding the Aβ aggregates. In addition, the level of acetylcholine in the brains of Tg2576 mice was considerably enhanced upon G-CSF treatment [28]. However, the five day course of G-CSF had no effect on brain amyloid load measured either immunohistochemically or by ELISA. In contrast, a two-week course of G-CSF administration to a double transgenic mouse model of AD (tg APP/PS1) resulted in a significant decrease in amyloid burden as well as improvement in cognitive performance in a radial arm water maze [29].

## G-CSF/G-CSF receptor

A promising target for the development of a novel anti-AD therapy is the G-CSF receptor and its ligand G-CSF. The natural human glycoprotein exists in 174 and 180 amino acid forms of approximately 19,600 grams per mole. The more abundant and active 174 residue form has been used in the development of pharmaceutical products by recombinant DNA technology. The three proprietary recombinant G-CSF proteins include lenograstim (Granocyte^®^), filgrastim (Neupogen^®^), and pegylated filgrastim (Neulasta^®^); the pegylation conferred a longer duration of action.

Although G-CSF has been used primarily as an agent to treat leukopenia, the agent has been studied in animal models of stroke where it has been reported to reduce brain damage and improve outcome [30-33]. Several ongoing clinical trials are evaluating the effectiveness and safety of G-CSF for treatment of ischemic stroke, the results of which are still pending [34]. It has also been used experimentally to treat myocardial infarction in humans [35-39]. In addition, G-CSF has putative neuroprotective activities independent of its regulation of granulocyte differentiation and stem cell mobilization [31,33].

G-CSF receptor, a member of the hematopoietin cytokine receptor superfamily, functions as a homodimer and requires the recruitment of cytosolic protein tyrosine kinases to transduce its signal [40]. At least two protein tyrosine kinases are primarily involved: Jak2, a member of the Janus family, and Lyn, a member of the Src family. Through poorly understood mechanisms, these kinases functionally interact with the G-CSF receptor. Jak2 primarily enlists members of the signal transducer and activator of transcription (STAT) family and Lyn phosphorylates a number of adaptor molecules linking the G-CSF receptor to phosphatidylinositol 3'-kinase and the extracellular signal-regulated kinase (Erk) pathways [40]. The characterization of the specific intracellular signaling pathways to distinct cell responses elicited by G-CSF is a major objective of current studies [40].

## Stem cell factor (c-kit ligand)

SCF is a glycoprotein (also known as 'steel factor' or c-kit ligand) that plays a key role in hematopoiesis, acting both as a positive and negative regulator, often in synergy with other cytokines. It also plays a pivotal role in mast cell development, gametogenesis, melanogenesis and neurogenesis. The SCF receptor, *c-kit*, is expressed in neural progenitors in cell cultures and in neuroproliferative zones of the adult rat brain. *In vivo *administration of SCF increased bromodeoxyuridine labeling of immature neurons in these regions [11]. When SCF was co-administered with G-CSF 10 days after an ischemic lesion in the mouse, the recovery of function and enhancement of neurogenesis was much greater than when either was given alone [16].

## Translation to clinical trials in Alzheimer's disease

Since G-CSF is already in advanced clinical trials for ischemic stroke [34], pre-clinical research with this HGF should be easily translated into therapeutic trials for AD. The initial approach would be to mobilize the patient's BM by administering drugs (HGFs such as G-CSF and/or SCF) that increase the infiltration of BM-derived cells into the brain where they can effectively reduce the amyloid load and promote other repair processes, including the formation of new neurons. Another approach would utilize the BM transplantation paradigm. AD patients would have an infusion of BM that is genetically engineered to deliver critical proteins that might enhance clearance of amyloid, provide neuroprotection, and stimulate neurogenesis. Eventually, a drug discovery screening program designed to target the G-CSF receptor should be able to find peptide-mimetics that interact with the G-CSF-receptor to produce the desired biological effects.

## Conclusion

The application of HGF for the treatment of AD has not yet been undertaken but there is a strong rationale for initiating clinical studies. In the past five years it has become evident that BM houses more 'primitive', multipotent stem cells that are capable of giving rise to tissues of all embryonic germ layers. BM-derived cells have been shown by independent investigators to give rise to neural cells *in vitro*, and *in vivo *studies have shown that BM cells migrate into the brain where they appear to differentiate into neurons and glia. The mechanism for transdifferentiation of BM to neural cells is not clear and may reflect the capacity of BM-derived cells to fuse with injured neurons. The *in vitro *demonstrations of transdifferentiation of BM cells has utilized different sub-populations of marrow and various combinations of cell culture media and growth factors. The combination of retinoic acid and BDNF were effective in driving a stromal BM cell population into neuron-like cells. More recently, a fragment of amyloid precursor protein has been reported to potentiate the nerve growth factor/retinoic acid-induced transdifferentiation of BM-derived adult progenitor cells into neural progenitor cells, and more specifically, enhances their terminal differentiation into a cholinergic-like neuronal phenotype. In *vivo *studies on the problem of transdifferentiation suggest that generation of new neurons from a bone marrow progenitor, if it really occurs, may be a rare phenomenon. Nevertheless, BM cells from the periphery are the source of at least 10% of the adult brain's immune cells (microglia) under normal conditions, and following various brain injuries, the trafficking of BM cells from blood to brain is markedly increased. Microglia have been clearly observed in the core of amyloid plaques in transgenic mouse models of AD. Perhaps as many as 10% of these cells originate from the BM and the Aβ-40 and Aβ-42 isoforms are able to trigger this chemoattraction. These newly recruited cells also exhibit a specific immune reaction to both exogenous and endogenous Aβ in the brain. G-CSF inhibits programmed cell death and promotes neurogenesis. Given that G-CSF administration in a mouse model of AD has been reported to improve performance in a hippocampal-dependent learning task and administration of G-CSF has been shown to be safe and well-tolerated in early clinical studies in patients with ischemic stroke, initiation of clinical trials to reverse dementia or forestall progression of AD is warranted.

## List of abbreviations used

Aβ: amyloid-β; AD: Alzheimer's disease; BDNF: brain-derived neurotrophic factor; BM: bone marrow; BMP: bone morphogenetic protein; G-CSF: granulocyte-colony stimulating factor; GFP: green fluorescent protein; HGF: hematopoietic growth factor; HSC: hematopoietic stem cell; NSC: neural stem cell; SCF: stem cell factor.

## Competing interests

The authors declare that they have no competing interests.

## Authors' contributions

Each author contributed to the concepts discussed and participated in preparation of the manuscript.
